# Functional expression of CCL8 and its interaction with chemokine receptor CCR3

**DOI:** 10.1186/s12865-017-0237-5

**Published:** 2017-12-28

**Authors:** Baosheng Ge, Jiqiang Li, Zhijin Wei, Tingting Sun, Yanzhuo Song, Naseer Ullah Khan

**Affiliations:** Center for Bioengineering and Biotechnology, China University of Petroleum (East China), Qingdao, 266580 People’s Republic of China

**Keywords:** Agonist, CCL8, Chemokine, Chemokine receptor CCR3, Expression, Interaction

## Abstract

**Background:**

Chemokines and their cognate receptors play important role in the control of leukocyte chemotaxis, HIV entry and other inflammatory diseases. Developing an effcient method to investigate the functional expression of chemokines and its interactions with specific receptors will be helpful to asses the structural and functional characteristics as well as the design of new approach to therapeutic intervention.

**Results:**

By making systematic optimization study of expression conditions, soluble and functional production of chemokine C-C motif ligand 8 (CCL8) in *Escherichia coli* (*E. coli*) has been achieved with approx. 1.5 mg protein/l culture. Quartz crystal microbalance (QCM) analysis exhibited that the purified CCL8 could bind with C-C chemokine receptor type 3 (CCR3) with dissociation equilibrium constant (*K*_D_) as 1.2 × 10^−7^ M in vitro. Obvious internalization of CCR3 in vivo could be detected in 1 h when exposed to 100 nM of CCL8. Compared with chemokine C-C motif ligand 11 (CCL11) and chemokine C-C motif ligand 24 (CCL24), a weaker chemotactic effect of CCR3 expressing cells was observed when induced by CCL8 with same concentration.

**Conclusion:**

This study delivers a simple and applicable way to produce functional chemokines in *E. coli*. The results clearly confirms that CCL8 can interact with chemokine receptor CCR3, therefore, it is promising area to develop drugs for the treatment of related diseases.

**Electronic supplementary material:**

The online version of this article (10.1186/s12865-017-0237-5) contains supplementary material, which is available to authorized users.

## Background

Chemokines are a class of structurally related chemotactic cytokines, which along with their cognate receptors can regulate a variety of cellular functions, including immunodeficiency virus type I infection, cancer metastasis, arthritis, asthma and neurodegenerative diseases [1, 2]. Chemokines and their derived antagonists have been demonstrated as an effective way for treatment of allergic diseases [3, 4]. Therefore, chemokines are attracting more and more interests in therapeutic potential and extensively demonstrated in the pharmaceutical development [5, 6].

Despite the simplicity of these small cytokines, structural and functional studies of chemokines are far more complicated than initially expected [2]. Firstly, it is challenging to obtain functional chemokines in considerable amount (milligram) from natural sources [7]. Over-expression of chemokines in *E. coli* can be achieved, but tends to form inclusion bodies [8], where refolding of chemokines are time consuming and costly effective [9]. Secondly, due to the promiscuity of the chemokine system, the chemokine receptor can bind with various chemokines, such as C-C chemokine receptor type 1 (CCR1) can bind with chemokine C-C motif ligand 3 (CCL3), chemokine C-C motif ligand 5 (CCL5), chemokine C-C motif ligand 7 (CCL7) and chemokine C-C motif ligand 23 (CCL23). Conversely, one chemokine can also interact with different receptors, for example CCL5 (Rantes) can interact with CCR1, CCR3 and C-C chemokine receptor type (CCR5) [10, 11], inducing different responses [12]. The crosstalking of chemokines and chemokine receptors are still not clear so far [1, 13]. Therefore, developing an efficient method for functional expression of chemokines and their clear interactions with receptors will be helpful for designing new medicines with high efficacy and low side effects [3].

CCL8 (monocyte chemotactic protein-2, MCP-2) belongs to the CC chemokine sub-family [1], which has been reported as an agonist of C-C chemokine receptor type 2 (CCR2) and CCR5 [14], and plays a pivotal role in the control of leukocyte chemotaxis, HIV entry and other inflammatory diseases [15–17]. Despite its important medical purpose, CCL8 as well as other chemokines tends to form inclusion bodies when overexpressed in *E. coli* [8, 18]. There is increasing demand for developing protocols to obtain milligrams quantity of soluble and functional CCL8 for biological studies and drug screening [19–21]. The cross interaction of CCL8 with another important allergic related chemokine receptor CCR3 [9, 22] are still highly controversial. Several reports in the literature indicate that CCL8 is one of the potential agonist of CCR3 [1, 6, 23], while others are not involved [2, 12, 14, 24]. To our knowledge, still no experimental reports exist on interactions of CCL8 with CCR3, and their detailed binding kinetics, thermodynamics and functional responses are still not well characterized.

Here, a simple and efficient protocol for soluble and functional production of CCL8 in *E. coli* was established. Binding assay of CCL8 with CCR3 in vitro was carried out using QCM method. Internalization and chemotaxis of CCR3 expressing cells induced by CCL8 were also characterized. Our work provides an efficient way to produce functional chemokines in *E. coli*, and provides the detailed interaction informations of CCL8 with CCR3 for potential drug developemnt and treatment of related diseases.

## Results

### Optimization of expression conditions

**Fig. 1:**
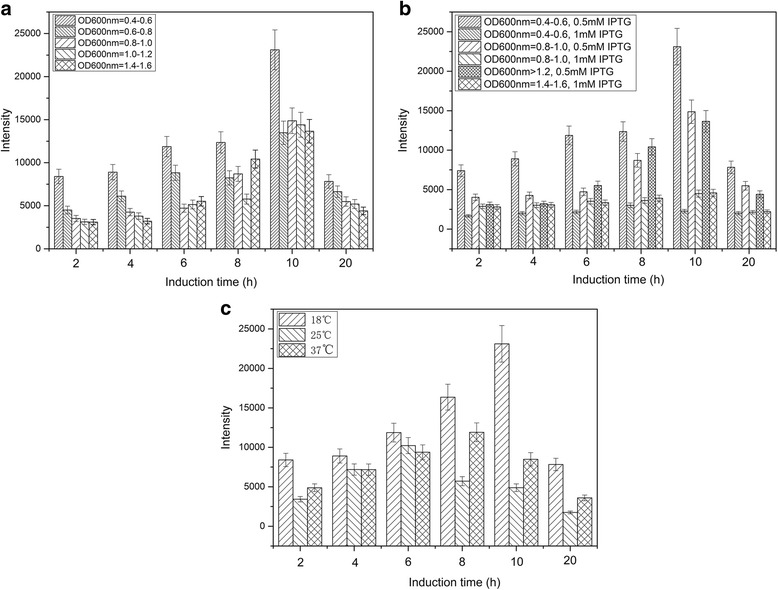
Optimization of induction phase, inducer concentration and induction temerature. Production of CCL8 was characterized using dot-blot. The intensities of dot-blot were averaged and error bars were calculated based on three times experiments. **a** Effect of induction phase on the expression of pET28a-CCL8. **b** Effect of inducer concentration on the expression of pET28a-CCL8. **c** Effect of expression temperature on the expression of pET28a-CCL8

To achieve soluble production of CCL8, the culture conditions of *E. coli* harboring pET28 a-His_12_-CCL8 plasmid were systematically optimized, such as culture temperature, induction phase and induction concentrations. The induction phase plays an important role for soluble production of CCL8. As shown in Fig. 1a, the best induction phase is found as induction at OD_600nm_ of 0.4-0.6 when induced with 0.5 mM Isopropyl β-D-Thiogalactoside (IPTG). Later or earlier induction would significantly result in lower yield of soluble aimed proteins. As biologically active cytokine, the accumulation of CCL8 would be harmful for *E. coli* cells. Therefore, total induction time can also obviously influence yield of soluble CCL8 (Fig 1a). It is observed that within 10 h, the cell density increased with increasing culture time. However, after that the cell density decreased as time prolonged, resulting in lower yield of soluble proteins. Furthermore, culture temperature and concentration of inducer also influence the growth of *E. coli* cells and show obvious effects on soluble production of CCL8 (Fig. 1b and c). After optimization, maximum production of soluble CCL8 was obtained when induced by 0.5 mM IPTG at OD_600nm_ as 0.4-0.6, with induction temperature as 18°C for totally 10 h induction. The totally expression level of His_12_-tagged CCL8 fusion protein was determined as 12-16 mg protein/l culture using dot blot method where the purified chemokine CCL11 was used as a standard.

### Purification and characterization of CCL8

**Fig. 2:**
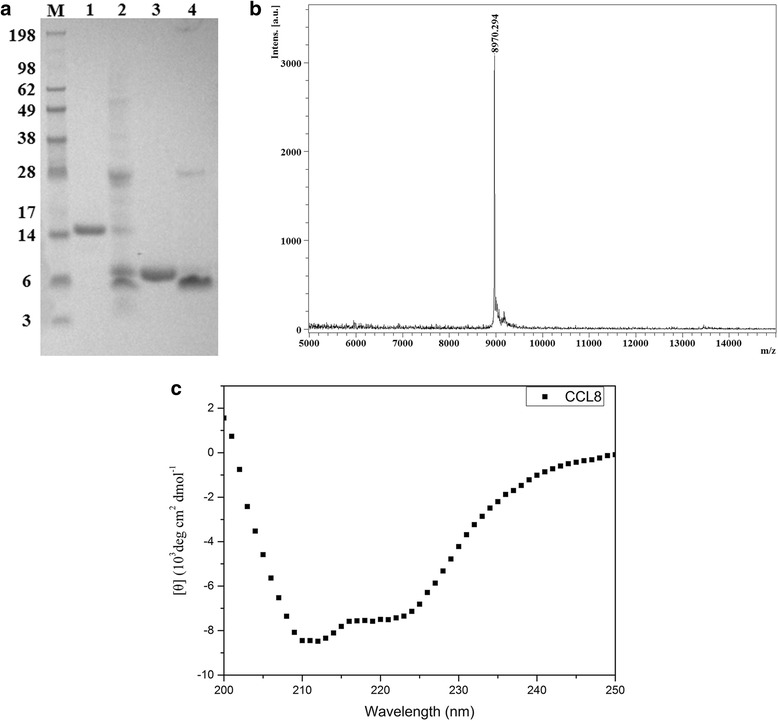
SDS-PAGE and circular dichroim analysis of CCL8 after digestion. **a** SDS-PAGE analysis of CCL8. Lane M represents protein standard markers. Lane 1 is His-tagged CCL8 fusion protein; lane 2, digested mixture of His-tagged CCL8 fusion protein; lane 3, purified CCL8 protein after the second Ni affinity chromatography; and lane 4 represents the mixture of undigested fusion protein, fusion part of CCL8 and His_6_-TEV enzymes captured on column, respectively. **b** Mass spectrometry analysis of the purified CCL8. **c** Circular dichroism analysis of recombinant CCL8

Six liters of *E. coli* cells containing pET28a-His_12_-CCL8 plasmid were cultured at optimized conditions and then harvested by centrifugation. After cell lysis, the soluble His_12_-tagged CCL8 fusion protein was purified via Ni^2+^ affinity chromatography. The eluted protein shows a distinct band at 14 kDa, which is consistent with the theoretical molecular weight of His_12_-tagged CCL8 fusion protein. The result was further confirmed by western blot analysis using anti-6 × His tag monoclonal antibodies. The yield of His_12_-tagged CCL8 fusion protein was determined as 5-8 mg/l culture with purity over 90%, and the averaged recovery rate is about 50% in fusion protein purification. The fusion protein was then digested by His_6_-TEV (Tobacco Etch Virus) enzyme to remove the His_12_-tag fusion part. After that, the digested mixture was passed through another Ni^2+^ affinity chromatography column, on which the His_12_-tagged fusion part and His_6_-TEV were captured, and CCL8 without a tag was recovered as flow-through. The apparent molecular weight of purified CCL8 on the SDS-PAGE (sodium dodecyl sulfate polyacrylamide gel electrophoresis) was 8.9 kDa (Fig. 2a), which is agree with its theoretical molecular weight. The result was further confirmed with mass spectrometry analysis (Fig. 2b). The final yield of CCL8 after digestion and purification was about 1.5 mg protein/l culture, corresponding to a recovery yield of 19-30% from fusion proteins. The secondary structure of CCL8 was analyzed using circular dichroism (Fig. 2c), which exhibit a mixture of α helix and β-sheets, similar as other typical chemokines [1, 22]. The secondary structure content of the purified CCL8 was estimated on Dicroweb as 22% α helix and 18% β-sheets, which is well consistent with the 25% α helix and 20% β-sheets of crystal structure analysis [14]. It suggested that the purified CCL8 has been well folded into a reasonable structure.

### Binding assay of recombinant CCL8 with CCR3

**Fig. 3:**
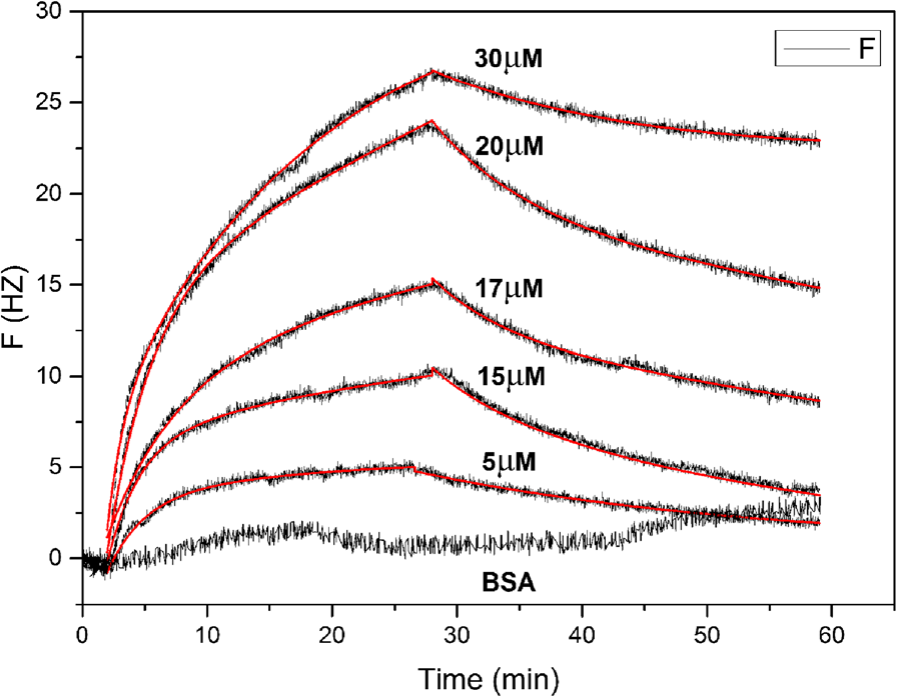
QCM sensorgrams for binding of different concentrations of CCL8 with CCR3. The experimental curves are shown in black, while the fitted curves are in red. The sensorgrams were fitted globally with a 1:1 binding model [22]

In order to determine whether CCL8 bind with CCR3 in vitro, the binding assay of CCL8 with chemokine receptor CCR3 was carried out using QCM method. From Fig. 3, typical QCM binding and dissociation curves are clearly observed compared with the control, suggesting that CCL8 can bind with CCR3 in vitro. By fitting these curves, the *K*_D_ value between CCR3 and CCL8 was obtained as 1.2 × 10^−7^ M, which is comparable to native agonists of CCR3, i.e. CCL11 and CCL24. They bind with CCR3 with *K*_D_ as 3.7 × 10^−7^ M and 3.0 × 10^−7^ M, respectively [22]. However, the association rate *k*_a_ and disassociation rate *k*_d_ values for CCL8 binding with CCR3 are 8.6 × 10^4^ M^−1^ s^−1^ and 0.00106 s^−1^ respectively (Table 1), which is much slower than those of CCL11 and CCL24 (Table 1). These results suggest that CCL8 is a potential agonist of CCR3 with similar binding affinity as CCL11 and CCL24, but slower kinetic binding rate [22]. It should be noted that the data obtained for CCR3 binding with CCL11 and CCL24 were determined using surface plasma resonance (SPR) methods, while the data for CCR3 binding with CCL8 were characterized using QCM, therefor it cannot be excluded that the difference of *K*_D_ value was generated from the two different methods.

**Table 1:** The thermodynamic and kinetic constants for CCR3 binding with different ligands

Agonists	*k*_a_(M^−1^ s^−1^)	*k*_d_(s^−1^)	*K*_D_ (M)
CCL8	8.6 × 10^4^	0.00106	1.2 × 10^−7^
CCL11^a^	2.0 × 10^5^	0.0740	3.7 × 10^−7^
CCL24^a^	6.3 × 10^5^	0.1900	3.0 × 10^−7^

^a^Data were taken from (Wang et al. [22])

### Internalization assay

**Fig. 4:**
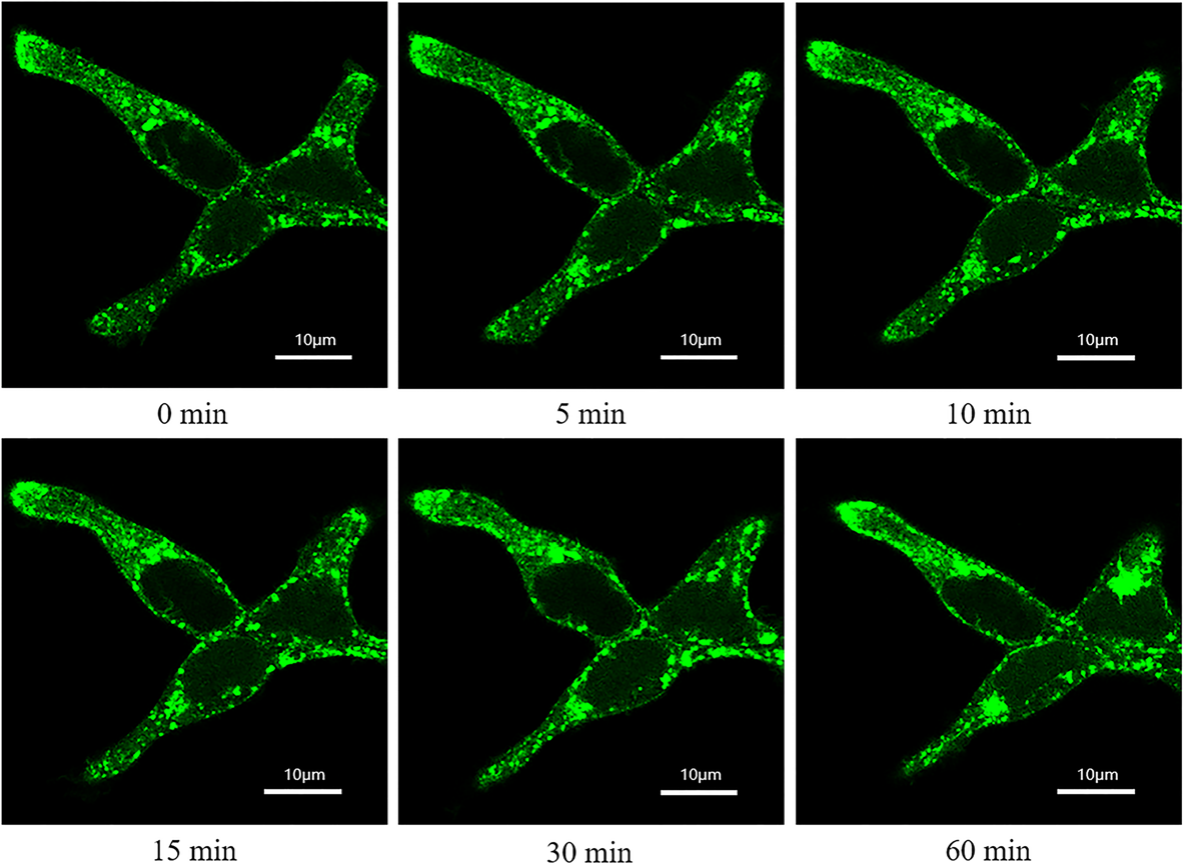
Internalization of CCR3 induced by CCL8. Cells were imaged at different time after stimulated by 100 nM CCL8 using a Nikon A1 confocal microscopy

Internalization of GPCR’s has been considered as a possible way of receptor desensitization after agonist stimulation [25]. The induced internalization of CCR3 with CCL8 on the cell surface was assessed using the TRx-HEK293 cells (Thermo Fisher) stably transfected with CCR3. The cells were treated with 100 nM CCL8, and the internalization process of CCR3 was imaged on a Nikon A1 confocal microscopy. After 30 min stimulation, CCR3-EGFP protein (enhanced green fluorescent) tended to form large aggregates and transferred into cytosol, which means that obvious internalization of CCR3 could be induced when treated with CCL8 (Fig 4).

### Chemotaxis test

**Fig. 5:**
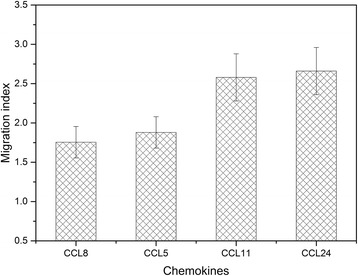
Chemotactic index of HEK293 cells stably transfected with CCR3 induced with different agonists. Errors bars were taken from three times repeats

Chemotaxis assays are useful tools for the evaluation of chemotactic ability of agonists. To explore the chemotactic ability of CCL8 inducing CCR3 expressing cells, chemotaxis test was carried out on Transwell chambers using TRx-HEK293 cells stably transfected CCR3-EGFP. From Fig. 5, it can be seen that CCL8 induce obvious chemotactic migration of TRx-HEK293 cells stably transfected CCR3, enabled that CCL8 can interact with CCR3 in vivo, confirms that CCL8 is one of agonists of CCR3. Compared with other agonists of CCR3, the chemotactic index of CCL8 is similar to CCL5, but lower than those of CCL11 and CCL24 (Fig. 5). The results obtained from the binding assay in vitro shows that CCL8 has similar binding affinity but slower binding and dissociation rate with CCR3 than those of CCL11 and CCL24, which may be an agreement for its weaker chemotactic index than CCL11 and CCL24.

## Discussion

Chemokines are clinically significant proteins, their structural and functional studies have attracted more and more interests nowadays [26, 27]. To obtain a high concentration of biological active chemokines in milligram with high purity is challenging tasks because of its naturally low yield and the formation of inclusion bodies when overexpressed in *E. coli* [8]. There are normally two ways to produce large amount of chemokines: one is overexpression of chemokines as inclusion bodies and then refolded them from denatured conditions [8, 28]. Another approach is expressing chemokines as fusion protein with different fusion tags, such as maltose binding protein (MBP), glutathione-S-transferase (GST) [19], and NusA [29]. Although this fusion chemokines have been proved to retain their binding activity with their cognate receptors, but to exclude any possible nonspecific interactions, the fusion part always need to be removed by exogenous enzyme digestion and further chromatography methods. The two methods mentioned above are usually low yield, time consuming and costly. By systematical efforts on optimization of expression conditions, the formation of inclusion bodies can also be significantly decreased, and soluble and functional expression of cytokines can be achieved [22, 30]. Our previous study has demonstrated soluble production of human chemokines CCL11 and CCL24, and here we present the protocol for soluble production of biologically active CCL8. We found that, although chemokines have similar tertiary structure and functions, but different optimal expression conditions are needed to achieve high level soluble production. For example, CCL5 (RANTES) cannot achieve milligram production of soluble and functional protein even with similar methods, suggested that it is necessary to develop a different protocol for production of various chemokines.

Chemokines participate in leukocyte trafficking and function, implicated in health and disease. Heterophilic interactions between chemokines and chemokine receptors may modulate their activities. However, systematic evaluation of interactions between chemokines and their specific receptors has not been established [31]. Human CCR3 (hCCR3) and CCL8 both belong to the CC chemokine-chemokine receptor sub-family [1], and plays pivotal roles in the control of leukocyte chemotaxis, HIV entry and other inflammatory diseases [15–17]. Unfortunately, the interaction of CCR3 and CCL8 is still not well characterized, therefore highly debated. Here using QCM binding assay as well as internalization and chemotaxis tests, we confirmed that CCL8 is one of the agonist of chemokine receptor CCR3, and their detailed kinetic and thermodynamic binding constants in vitro and in vivo were also characterized. Compared with other ligands of CCR3, CCL8 shows similar interaction with CCR3 as the universal ligands CCL5, but different from the native ligands of CCR3, such as CCL11 and CCL24. Our results provide important information for CCL8-CCR3 interaction system. These facts wukk deliver a great advantage in potential drug design and treatment of related diseases.

## Conclusions

A simple and reliable method for soluble expression of chemokine CCL8 has been established with a yield of 1.5 mg protein /l culture. The purified CCL8 can bind with chemokine receptor CCR3 in vitro with *K*_D_ value as 1.2 × 10^−7^ M, which is similar as CCL11 and CCL24. Obvious internalization and chemotactic migration of CCR3 expressing cells can be observed when induced with CCL8, which confirmed that CCL8 is one of the agonist of chemokine receptor CCR3. The antagonist development based on CCL8 will be a useful tool for development of efficient drugs and treatment of related diseases.

## Methods

### Recombinant plasmid construction

Gene of human CCL8 (NCBI locus: 1ESR_A) was commercially synthesized by GenScript Bio-company (China) with optimized codon usage for over- expression in *E. coli*. The gene was amplified by PCR using a forward primer as 5’-AGTGGATCCCATCATCACCATCACCACG AAAACCTGTATTTTCAGGGTCAACCGGATAGCGTGAGCATC -3′ and reverse primer as 5’-ACTAAGCTTTTATTACGGTTTCAGATTCTGAAAAATCTGATC C AG-3′. The PCR product were digested with *Bam*H I and *Hin*d III, and then ligated into pET28a vector (Novagen, USA) at the downstream of a T7 promoter. To facilitate downstream purification, another 6 × His tag was added onto the N terminus of CCL8 gene, resulting in pET28a-His_12_-CCL8 plasmid. A TEV enzyme digestion site was also inserted between the 6 × His tag and CCL8 gene for removal of any fusion part after purification. After confirmed by DNA sequencing, the recombinant plasmid was then transformed into *E. coli* strain BL21 (DE3) (Novagen) for optimization and protein expression.

### Optimization of expression conditions

In order to achieve maximal soluble production of CCL8, the expression conditions of cells harboring pET28a-His_12_-CCL8 were optimized systematically using a similar method as previously reported [22]. Briefly, colonies from the transformation plates were cultured in 5 ml Luria-Bertani broth (LB) medium overnight at 37°C with shaking. 1 ml of overnight culture was then inoculated into 100 ml of fresh Terrific Broth (TB) medium supplemented with 50 μg/ml kanamycin, and IPTG were applied to induce overexpression of target proteins. The induction phase, concentration of inducer and induction temperature were optimized by inducing at OD_600nm_ as 0.4-0.6, 0.6-0.8, 0.8-1.0, 1.0-1.2 and 1.4-1.6, and IPTG concentrations of 0.1 mM, 0.2 mM, 0.3 mM, 0.5 mM and 1 mM and induction temperature as 18°C, 25°C and 37°C, respectively. After induction with IPTG, samples were taken at regular times, and then lyzed and centrifuged at 12000 g for 10 min at 4 °C. Supernatant were then taken and analyzed using dot-blot with mouse anti-His monoclonal antibody as primary antibody and HRP (horse radish peroxidase)-labeled goat anti-mouse antibody as secondary antibody. The blot was finally stained using the Amersham ECL plus, and then detected on a FLA-5100 imaging system (Fuji, Japan). The intensity of each dot was analyzed with MultiGauge Ver.3.X software (Fuji, Japan) according to manufacturer’s instructions (Additional file 1: Figure S1).

### Expression and purification of CCL8 in *E. coli*

Six liters of *E. coli* cells were harvested and resuspended in phosphate buffered saline (PBS) buffer. After broken on an ultra-high pressure homogenizer*,* the crude extract was obtained by centrifugation at 12,000 g for 20 min at 4 °C, and then applied to a HiTrap™ Chelating HP column (GE Healthcare). The impurities were washed off with PBS buffer containing 250 mM imidazole (pH 7.4) and the target CCL8 fusion protein was eluted with PBS buffer containing 500 mM imidazole (pH 7.4). The eluent was then buffer exchanged into 1 × PBS, and 6 × His-tagged TEV enzymes (plasmids expressing TEV enzymes was obtained as courtesy from Alan Fersht’s lab at LMB, UK) were added for digestion of His_12_-CCL8 fusion proteins. The digestion mixture was passed through another HiTrap™ Chelating HP column, and the cleaved fusion part with His_12_-tag and the His_6_-TEV enzyme will be captured on the column, and CCL8 is recovered in the flow-through fraction. The purified CCL8 was collected and characterized using SDS-PAGE, mass spectrum and circular dichroism spectroscopy respectively.

### Quartz crystal microbalance measurement

The binding assay of CCL8 with CCR3 in vitro was carried out on a QCM-Z500 (Biolin Scientific, Sweden) instrument using Q-sense His tag capturing sensor at 25 °C. The sensor was first activated with 0.5 mM NiCl_2_. The 6 × His tagged CCR3 purified from stably transfected HEK293 cells was prepared as our previously reported [9] and immobilized on the sensor. Different concentrations of CCL8 were flow through the sensor respectively, and the binding of CCL8 to the immobilized CCR3 was monitored in real time with a flow rate of 50 μl/min. 5 μM BSA (bovine serum albumin) was used as control to exclude any possible non-specific interaction. The sensorgrams were fitted globally with a 1:1 binding model [22].

### Internalization assay

The TRx-HEK293 cells stably transfected CCR3-EGFP previously constructed in our lab [9] was used for the receptor internalization assay. The cultured cells were cultured in Dulbecco’s Modified Eagle Medium (DMEM) medium containing 0.5% bovine serum, and before internalization assay, cells was washed with HEPES buffer, and then stimulated with 100 nM CCL8. Images of CCR3-EGFP on cell surface before and after stimulation were captured by a confocal microscopy (Nikon A1, Japan) at different incubation times.

### Chemotaxis test

TRx-HEK293 cells stably transfected with CCR3-EGFP [9] were resuspended (1 × 10^4^) in DMEM containing 0.5% (*v*/v) bovine serum albumin. 100 μl cell suspensions were placed in the upper wells of Transwell chambers (Corning, USA) containing bare filter with a pore size of 8 μm. The CCL8 (100 nM) in 500 μl same medium was placed in the lower chambers. After incubation at 37 °C for 4 h, cells migrated though the filter were stained with crystal violet and counted by image J software. Migration index is obtained as values of migrated cell numbers induced with chemokines divided by migrated cell numbers without chemokines.

## Additional file

Additional file 1: Figure S1. Optimization of induction phase. Production of CCL8 was characterized using dot-blot. The intensities of dot-blot were averaged and error bars were calculated based on three times experiments. (A) Dot-blot images for optimization of induction phase. (B) Histogram for effect of induction phase on the expression of pET28a-CCL8. (TIFF 1708 kb)

## Abbreviations

BSA: bovine serum albumin
CCL11: Chemokine C-C motif ligand 11
CCL23: Chemokine C-C motif ligand 23
CCL24: Chemokine C-C motif ligand 24
CCL3: Chemokine C-C motif ligand 3
CCL5: Chemokine C-C motif ligand 5
CCL7: Chemokine C-C motif ligand 7
CCL8: Chemokine C-C motif ligand 8
CCR1: C-C Chemokine receptor type 1
CCR2: C-C Chemokine receptor type 2
CCR3: C-C Chemokine receptor type 3
CCR5: C-C Chemokine receptor type
DMEM: Dulbecco’s Modified Eagle Medium
*E. coli*: *Escherichia coli*
EGFP: enhanced green fluorescent protein
GST: glutathione-S-transferase
HRP: horse radish peroxidase
IPTG: Isopropyl β-D-Thiogalactoside
*K*_D_: Dissociation equilibrium constant
LB: Luria-Bertani broth
MBP: maltose binding protein
PBS: phosphate buffered saline
QCM: Quartz crystal microbalance
SDS-PAGE: sodium dodecyl sulfate polyacrylamide gel electrophoresis
SPR: surface plasma resonance
TB: Terrific Broth
TEV: Tobacco Etch Virus
