# Supplementary figures and images for: Functional expression of CCL8 and its interaction with chemokine receptor CCR3
Source: BMC Immunol. 2017 Dec 28;18:54. doi: 10.1186/s12865-017-0237-5 (PMC5745793; doi:10.1186/s12865-017-0237-5)

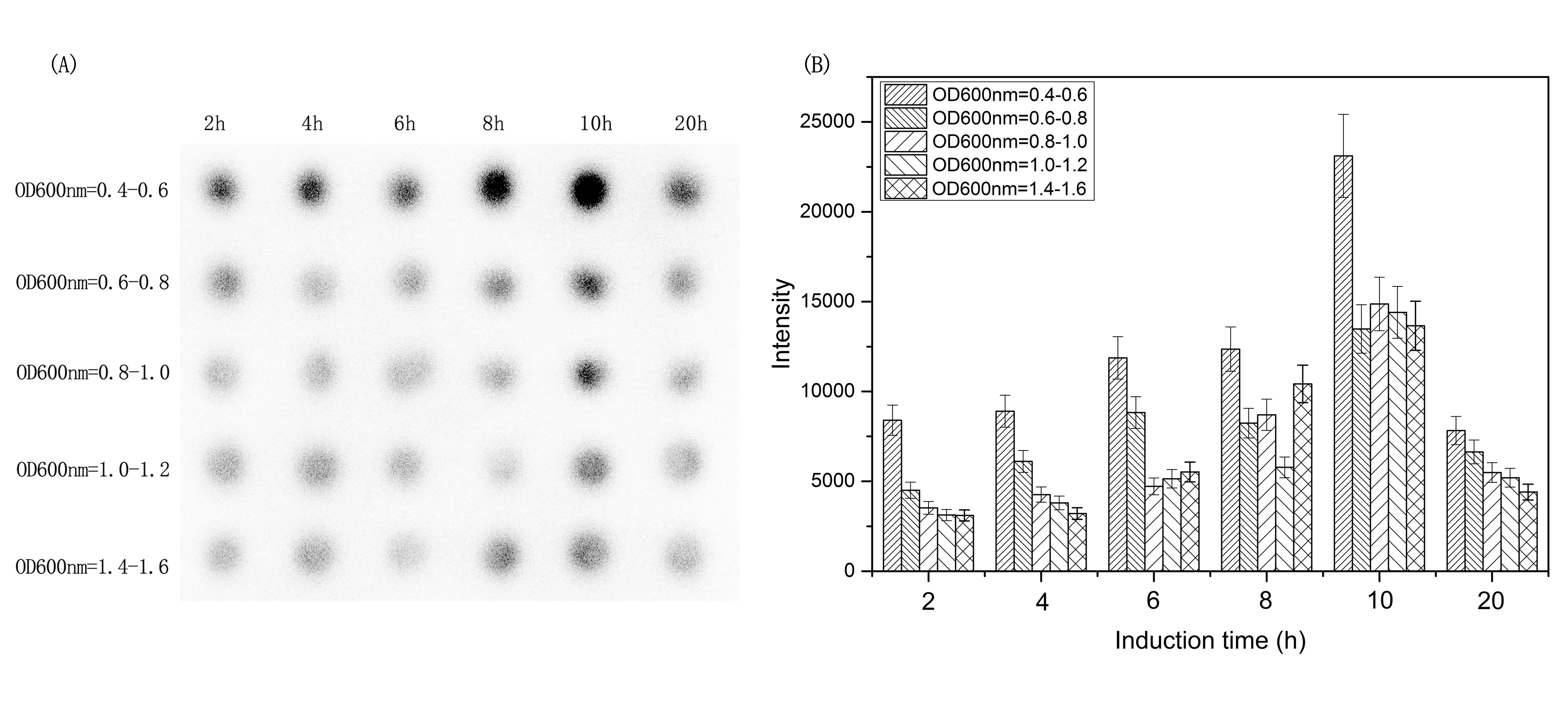

Supplement: Additional file 1: Figure S1. — Optimization of induction phase. Production of CCL8 was characterized using dot-blot. The intensities of dot-blot were averaged and error bars were calculated based on three times experiments. (A) Dot-blot images for optimization of induction phase. (B) Histogram for effect of induction phase on the expression of pET28a-CCL8. (TIFF 1708 kb) [file 12865_2017_237_MOESM1_ESM.tif]
